# Supplementary material for: The interactome of intact mitochondria by cross-linking mass spectrometry provides evidence for coexisting respiratory supercomplexes
Source: Mol Cell Proteomics. 2017 Dec 8;17(2):216–32. doi: 10.1074/mcp.RA117.000470 (PMC5795388; doi:10.1074/mcp.RA117.000470)
Supplement: Supplemental Data [file supp_RA117.000470_133922_0_supp_23978_fzffwf.zip › spectra_annotation/mito_spectra_annotation/406-1-12-1-7-1.pdf]

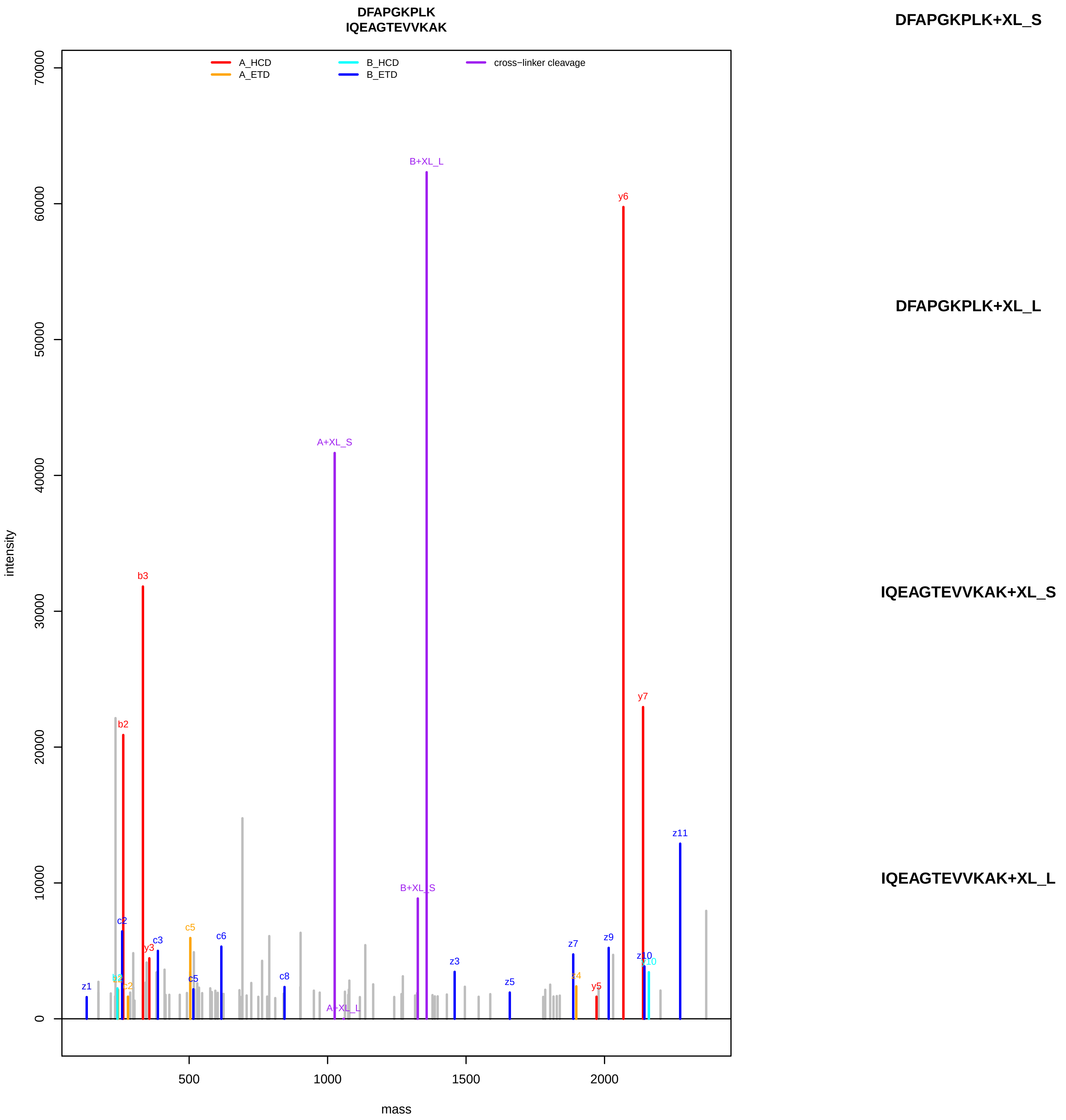
DFAPGKPLK
IQEAGTEVVKAK
A_HCD
A_ETD
B_HCD
B_ETD
cross−linker cleavage
intensity
mass
0
10000
20000
30000
40000
50000
60000
70000
500
1000
1500
2000
B+XL_L
y6
A+XL_S
b3
b2
y7
z11
B+XL_S
c2
c5
c6
c3
y3
z7
z9
z10
y10
z3
c8
c5
b2
c2
z1
z5
z4
y5
A+XL_L
DFAPGKPLK+XL_S
DFAPGKPLK+XL_L
IQEAGTEVVKAK+XL_S
IQEAGTEVVKAK+XL_L
